# Low-Density Lipoproteins Increase Proliferation, Invasion, and Chemoresistance via an Exosome Autocrine Mechanism in MDA-MB-231 Chemoresistant Cells

**DOI:** 10.3390/biomedicines12040742

**Published:** 2024-03-27

**Authors:** César Y. Castañeda-Sánchez, Brenda Chimal-Vega, Roberto León-Gutiérrez, Adrián Ernesto Araiza-Robles, Nicolás Serafín-Higuera, Angel Pulido-Capiz, Ignacio A. Rivero, Raúl Díaz-Molina, Manuel Alatorre-Meda, Eustolia Rodríguez-Velázquez, Victor García-González

**Affiliations:** 1 Departamento de Bioquímica, Facultad de Medicina Mexicali, Universidad Autónoma de Baja California, Mexicali 21000, Mexico; yahel.castaneda@uabc.edu.mx (C.Y.C.-S.); brenda.chimal@uabc.edu.mx (B.C.-V.); roberto.leon@uabc.edu.mx (R.L.-G.); adrian.araiza@uabc.edu.mx (A.E.A.-R.); pulido.angel@uabc.edu.mx (A.P.-C.); rauldiaz@uabc.edu.mx (R.D.-M.); 2 Laboratorio Multidisciplinario de Estudios Metabólicos y Cáncer, Universidad Autónoma de Baja California, Mexicali 21000, Mexico; 3 Facultad de Odontología Mexicali, Universidad Autónoma de Baja California, Mexicali 21000, Mexico; nserafin@uabc.edu.mx; 4 Centro de Graduados e Investigación en Química, Tecnológico Nacional de México, Instituto Tecnológico de Tijuana, Tijuana 22510, Mexico; irivero@tectijuana.mx; 5 Centro de Graduados e Investigación en Química-Grupo de Biomateriales y Nanomedicina, CONAHCYT-Tecnológico Nacional de México, Instituto Tecnológico de Tijuana, Tijuana 22510, Mexico; manuel.alatorre@tectijuana.edu.mx; 6 Facultad de Odontología, Universidad Autónoma de Baja California, Tijuana 22390, Mexico; eustolia.rodriguez@uabc.edu.mx; 7 Centro de Graduados e Investigación en Química-Grupo de Biomateriales y Nanomedicina, Tecnológico Nacional de México, Instituto Tecnológico de Tijuana, Tijuana 22510, Mexico

**Keywords:** triple-negative breast cancer, chemoresistance, LDL, doxorubicin, exosomes, Hsp90, Hsp70

## Abstract

Dyslipidemias involving high concentrations of low-density lipoproteins (LDLs) increase the risk of developing triple-negative breast cancer (TNBC), wherein cholesterol metabolism and protein translation initiation mechanisms have been linked with chemoresistance. Doxorubicin (Dox) treatment, a member of the anthracycline family, represents a typical therapeutic strategy; however, chemoresistance remains a significant challenge. Exosomes (Exs) secreted by tumoral cells have been implicated in cell communication pathways and chemoresistance mechanisms; the content of exosomes is an outcome of cellular cholesterol metabolism. We previously induced Dox resistance in TNBC cell models, characterizing a variant denominated as variant B cells. Our results suggest that LDL internalization in parental and chemoresistant variant B cells is associated with increased cell proliferation, migration, invasion, and spheroid growth. We identified the role of eIF4F translation initiation factor and the down-regulation of tumor suppressor gene PDCD4, an inhibitor of eIF4A, in chemoresistant variant B cells. In addition, the exomes secreted by variant B cells were characterized by the protein content, electronic microscopy, and cell internalization assays. Critically, exosomes purified from LDL-treated variant B cell promoted cell proliferation, migration, and an increment in lactate concentration. Our results suggest that an autocrine phenomenon induced by exosomes in chemoresistant cells may induce modifications on signaling mechanisms of the p53/Mdm2 axis and activation of p70 ribosomal protein kinase S6. Moreover, the specific down-regulated profile of chaperones Hsp90 and Hsp70 secretion inside the exosomes of the chemoresistant variant could be associated with this phenomenon. Therefore, autocrine activation mediated by exosomes and the effect of LDL internalization may influence changes in exosome chaperone content and modulate proliferative signaling pathways, increasing the aggressiveness of MDA-MB-231 chemoresistant cells.

## 1. Introduction

Data from GLOBOCAN 2020 estimate that there were 19.3 million new cancer cases and 10 million cancer-related deaths, and also predicted this to increase by 47% in 2040 [1]. Breast cancer (BC) can be classified depending on the cellular expression of estrogen receptors (ER) as Luminal A or B, or by the expression of the human epidermal growth factor 2 (HER2), as HER2+. According to this classification there is a fourth molecular subtype of breast cancer which does not express ER and HER2 that is named triple-negative breast cancer (TNBC); this classification helps physicians select the best chemotherapy treatment depending on the cellular phenotype. TNBC is known to be a highly aggressive subtype of BC due to the lack of receptor expression. For these patients, therapeutic options are reduced; the main systemic alternative is cytotoxic chemotherapy [2,3], such as anthracyclines, one of the reference drugs. Poor survival has been documented compared to other BCs [4,5,6]. The factors regulating chemoresistance in TNBC could be associated with alterations in the unfolded protein response (UPR) and potentiated by low-density lipoprotein (LDL) [7].

During the chemoresistance transition, modifications of the UPR and protein translation initiation could induce cellular proliferation, migration, and invasion, which are considered cancer hallmarks. In this regard, molecular, biochemical, and structural adaptations are triggered during the acquisition of chemoresistance in tumoral cells [7,8]. Likewise, the activation of antioxidant systems, expression of specific transporters, detoxifying enzymes, and adaptive mechanisms such as inhibition of apoptosis pathways (e.g., p53-MDM2) are critical in chemoresistance development. Doxorubicin (Dox) is an anthracycline drug that inhibits replication and transcription by interacting with DNA and blocking topoisomerase II, increasing ROS generation and promoting apoptosis. Furthermore, in cancer cells, modifications in targets regulating cholesterol metabolism such as de novo synthesis, endocytosis, and oxidation are contributing factors to cancer development [7] and potentially chemoresistance. Reports suggest several levels of association between LDL cholesterol and breast cancer risk [8,9,10]. For instance, based on the data of the Global Lipid Genetics Consortium and the Breast Cancer Association Consortium with >400,000 participants, Nowak and Arnlov (2018) reported that genetically raised LDL cholesterol is associated with a higher BC risk [11]. In this regard, intracellular cholesterol metabolism associated with high LDL concentrations must restrain several molecular mechanisms.

LDL hypercholesterolemia could trigger alterations of three UPR targets, IRE1, ATF6α, and PERK, which together promote mRNA degradation and increase the translation of chaperones, maintaining protein homeostasis [7,12]. Chaperones are able to regulate several growth signaling pathways [13], with implications in mechanisms of paracrine and autocrine cellular communication. Indeed, secreted factors by cancer cells compromise drug sensitivity [14]. Cancer cell-secreted chaperones could contribute to cancer progression in an autocrine way and correlate with the disease stage [15,16], inducing chemoresistance even more [14].

Eukaryotic initiation factors eIF4A, eIF4E, and eIF4G integrate the translation initiation complex eIF4F, which binds to the 7-methyl guanylate (m7G) of 5′mRNA, selectively modulating mRNA translation. A condition that suppresses the assembly of the eIF4F complex is the binding of eIF4A with programmed cell death protein 4 (PDCD4) [16]. PDCD4 activity has been associated with the inhibition of tumor cell proliferation and chemoresistance [17]. A PDCD4 regulatory mechanism is triggered by its degradation induced by the mTOR/p70 ribosomal protein kinase S6 (p70S6kα) pathway, a central modulator of cell growth, proliferation, survival, and differentiation in response to extracellular stimuli [18], possibly involving vesicles such as exosomes (Exs).

Exosomes delivered by tumor cells may play critical roles in the acquisition of chemoresistance [19]. These are lipid bilayer-limited vesicles associated with tumor development, promoting intercellular communication mechanisms [20]. Exosomes’ role in chemoresistance is broad and dependent on the type of cancer [20,21,22]. Furthermore, their function is influenced by drug treatment. For instance, exosomes derived from resistant tumor cells transport cargo molecules such as multidrug resistance protein 1, FAK, HER2, Akt, Wnt/β-catenin, and microRNAs [20,23,24]. In addition, the exosome secretion is associated with metabolic status of chemoresistant cells [25]. Therefore, through an autocrine pathway, cells can increase chemoresistance and cancer hallmarks such as migration, invasion, and cellular progression. In this regard, migration and invasion give rise to metastasis. Migration is associated with cell motility [26], wherein integrin proteins coordinate the extracellular matrix with the cytoskeleton; this process, in turn, promotes FAK activation through Y^397^ phosphorylation, which is associated with metastasis [27]. Likewise, FAK gene amplification and protein overexpression are connected with tumor progression, favoring migration, invasion, and metastasis [28]. The influence of LDL on the content of exosomes may be critical to these phenomena.

Cholesterol and ceramide levels in the plasma membrane, endoplasmic reticulum, and endosomal membranes could influence the content and size of exosomes [27]. Indeed, chaperones could be secreted via the exosome pathway [28]. Heat shock protein (Hsp) 90 and Hsp70 are the main chaperones transported. Hsp90α plasma levels have been considered a novel pan-cancer diagnosis biomarker [29]. Thus, Hsp90 levels in exosomes may represent a biomarker of tumoral progression [30], and alterations in plasmatic LDL cholesterol must modify these mechanisms. In the case of platinum sensitivity in ovarian cancer models, cholesterol homeostasis modulates the sensitivity [31]; resistant cells showed reduced cholesterol biosynthesis, and the uptake of exogenous cholesterol is critical [31]. Moreover, Hsp90 plays pivotal roles in cholesterol adaptations for drug resistance, and Hsp90 potentially facilitates cholesterol redistribution [6]. Therefore, an association between the function of Hsp90 and the role of LDL could be established.

Hsp90 and Hsp70 must be associated with the regulation of membrane receptor function considering the potential chaperone (Hsp90α) localization on the surface membrane of tumor-secreted exosomes [15]. Indeed, Hsp90 immunostimulatory role by the recognition of receptors such as CD91+ scavenger receptor [32] and a potential regulation on insulin receptor has been described [33]. Taken together, new mechanisms associated with chaperone-associated exosomes could be associated with chemoresistance.

Previous results suggest that PDCD4 down-regulation is a condition in doxorubicin chemoresistance in TNBC cells, modulating responses connected with the PERK pathway of UPR [17]. In this work, after treatment with LDL, exosomes derived from chemoresistant cells exert an autocrine phenomenon that induces cellular proliferation, migration, and invasion. The connection between LDL cholesterol overload on the PDCD4/eIF4A axis and a specific change in the profile of chaperone secretion in exosomes, down-regulated Hsp90-Hsp70, potentiates the malignancy phenotype in chemoresistant TNBC cells. Thus, LDL could promote the exosome release with a fingerprint of chaperone content.

## 2. Materials and Methods

### 2.1. Materials

Cell culture reagents were purchased from Thermo-Fisher (Carlsbad, CA, USA), and tissue culture plates and other plastic materials were obtained from Corning Inc. (Corning, NY, USA). Salts, buffers, and MTT were obtained from Merck (Darmstadt, Germany). Doxorubicin (99% of purity) was obtained from Merck (Darmstadt, Germany). Antibodies anti-XBP1s and anti-p-eIF4E were purchased from Abcam (Cambridge, UK). Anti-β-actin, anti-eIF4A, anti-PDCD4, anti-c-Jun, anti-MMP-9, anti-Nrf2, anti-Mdm2, anti-Flot-2, anti-CD63, anti-Hsp90, anti-Hsp70, anti-p53, anti-p70S6Kα, and anti-p-p70S6Kα were obtained from Sta. Cruz Biotechnology (Dallas, TX, USA).

### 2.2. Cell Culture

MDA-MB-231 cells corresponding to highly aggressive and invasive human mammary gland adenocarcinoma were characterized. The TNBC cell line MDA-MB-231 (American Type Culture Collection, ATCC, Manassas, VA, USA) was grown in DMEM medium supplemented with 10% fetal bovine serum (FBS), 10 U/mL penicillin, 10 µg/mL streptomycin, and 25 µg/mL amphotericin B. Cultures were maintained at 37 °C in a humidified atmosphere of 95% air and 5% CO_2_. The culture medium was changed every 3 to 4 days and also passed once per week, according to ATCC recommendations.

### 2.3. Chemoresistance Protocols

To generate drug-resistant cellular models, MDA-MB-231 cells were seeded at a density of 8 × 10^4^ cells/mL. In one protocol, throughout the cell treatment with Dox increasing concentrations (0–100 nM), a resistant variant was obtained during three months of treatment. Cells were cultured in a Dox-maintaining dose (15 nM) based on the protocol reported by Carlisi et al., 2017 [34]. In a second procedure, we evaluated a scheme of a lethal Dox dose (0.5 µM) for 24 h; later, the Dox treatment was diminished to 12.5 nM for 60 days and a recovery period of 30 days. This allowed for the generation of a variant denominated B. The third protocol performed a Dox-increasing dose for 60 days (15–60 nM range). Cell variants were evaluated by an MTT assay. This allowed us to generate three variants; however, most of the results were based on the variant denominated B. Untreated parental MDA-MB-231 cells were used in all experiments.

### 2.4. Isolation of LDL

Human plasma samples were obtained from a healthy donor that signed an informed consent, in order to obtain constant results. The protocol was performed according to the Declaration of Helsinki and approved by the Research Ethics Committee of the Facultad de Medicina Mexicali (FMM/CEI-FMM/006/2022-1). In the first step, plasma density was adjusted to 1.019 g/mL with KBr, and centrifuged at 345,500× *g* for 160 min using an S140-AT 2555 rotor; the layer containing VLDL and IDL was discarded. Later, the density was adjusted to 1.053 g/mL and centrifuged at 377,000× *g* for 200 min; the upper fraction containing LDL was recovered. The HDL fraction was isolated, adjusting to a 1.21 g/mL density with a centrifugation process of 377,000× *g* for 180 min. LDL fraction was dialyzed against 150 mM NaCl, filtered through 0.45 μm. LDL protein was measured with the bicinchoninic acid method (BCA). To evaluate the quality of the isolates, the content of the apolipoproteins apoB-100 and apoA1 was evaluated in the respective fractions by Western blot.

### 2.5. LDL Fluorescent Labeling

LDL fluorescent labeling was carried out with the dilC18 fluorescent probe (D3911), which is incorporated into the phospholipid monolayer, by incubating 10 µL of the probe (2 mg/mL) for each 1 mg of protein–LDL during 18 h at 37 °C, obtaining dil-LDL lipoproteins. To recover the fluorescent lipoproteins, the solution was adjusted to a 1.053 g/mL density and centrifuged at 377,000× *g* for 3 h. Finally, the fraction was recovered and dialyzed against PBS and filtered through 0.45 µm. This procedure was based on previous methods [35]. For experimental treatments in cell cultures, a fasting period was carried out.

### 2.6. Cell Viability

Cell viability was evaluated employing the MTT assay according to the previous protocol used in our group [36]. In this assay, a cellular density of 100,000 cells/mL was evaluated. In the last step, formazan crystals were dissolved in a lysis buffer containing SDS 20% and N,N-dimethylformamide 50% (pH 3.7) for 12 h at 37 °C. Optical density was measured at 570 nm using a microplate reader. In all experiments, control conditions were evaluated, and the same number of cells was maintained.

### 2.7. Black Sudan Staining

Cell cultures were incubated under several conditions of LDL concentrations (0–50 µg/mL) in a 20 mm well (100,000 cells/mL). Further to the treatments, cell cultures were washed with PBS and fixed with paraformaldehyde 2%, and stained with a Sudan Black B protocol (199664). The staining solution of Sudan Black B (500 mg) was prepared in acetone (20 mL). Then, the solution was transferred to an acetic acid solution (15% *v*/*v*). The solution was stirred vigorously for 60 min. To eliminate precipitate, the solution was centrifuged at 3500 rpm for 10 min.

### 2.8. Western Blotting (WB) Analysis

Cells proliferated until 90% of confluence, initially seeded at a density of 100,000 cells/mL in plates of 20 or 60 mm. Next, cells were incubated under indicated treatments. Cells were washed with PBS and lysed for 35 min at 4 °C with protein buffer lysis containing protease and phosphatase inhibitors. After centrifugation at 4100× *g* for 10 min, the supernatant was recovered, and protein quantification was performed by BCA. Samples (25 μg/lane) from the total protein fraction were analyzed by SDS-polyacrylamide gel electrophoresis (SDS-PAGE) on 8–12% gels according to the molecular weight of the targets, and were further transferred to PVDF membranes (Millipore, Burlington, MA, USA). Membranes were blocked with 5% nonfat milk in Tris-buffered saline 0.1% Tween-20 (TBS-T) for 1 h at 37 °C, and incubated overnight (4 °C) with the corresponding primary antibody: anti-XBP1s (1:500), anti-β actin (1:500), anti-eIF4A (1:500), anti-eIF4E (1:400), anti-PDCD4 (1:600), anti-MMP-9 (1:150), anti-Mdm2 (1:300), anti-FAK (1:200), anti-p53 (1:10,000), and anti-p-FAK397 (1:250). Following washing with TBS-T, membranes were further incubated for 2 h at 37 °C with the corresponding horseradish peroxidase-conjugated secondary antibodies. Next, membranes were washed with TBS-T and HRP activity was detected with the Immobilon Western kit (Millipore, Burlington, MA, USA). Analysis of immunoblots was carried out with the ImageJ program, and the figures generated show the representative blots.

### 2.9. Immunoprecipitation Assays

eIF4E/eIF4A complexes were evaluated by co-immunoprecipitation assays under different treatments. For these immuno-precipitation assays, cellular fractions (300 µg) were incubated with the antibody anti-eIF4A (1:400) for 2 h at 4 °C. Then, immune complexes were precipitated with protein G Agarose Fast Flow (Millipore) 12 h at 4 °C, according to the previous protocol [36]. Immuno-precipitated proteins were washed 3 times, and suspended in Laemmli buffer, separated by SDS-PAGE gels and transferred to PVDF membranes for WB analysis. eIF4E detection was performed based on a previously described protocol.

### 2.10. Doxorubicin Internalization (Dox)

Dox concentrations in the extracellular medium were evaluated, both in parental and resistant cells. Culture media were recovered and subsequently centrifuged at 5000 rpm for extracellular quantification to eliminate the cell debris. Samples were processed in a Cary Eclipse fluorescence spectrophotometer (Agilent Technologies, Inc., Santa Clara, CA, USA), using a λExc of 470 nm and λEm of 595 nm [37]. This procedure was carried out in 20 mm plates under cell cultures seeded at a density of 100,000 cells/mL.

In another experimental panel, under the same cellular density, MDA-MB-231 cultures at 90% confluence were incubated after 2 h of fasting, and then treated under different conditions with Dox in the OptiMEM medium (Thermo Fisher Scientific, Waltham, MA, USA). After carrying out the experiments in 10 mm wells, cells were washed twice with PBS and recovered in a volume of 200 µL. Cell characterization was performed with a Beckman–Coulter cytometer Cytoflex (Brea, CA, USA), and 30,000 events were registered.

### 2.11. Scratch-Wound Assay

Cells seeded at a density of 100,000 cells/mL and reaching a confluency of 90% were treated for 2 h of starvation; then, cultures were washed twice with PBS. Next, cultures were treated with 12 μM mitomycin-C for 2 h, and the culture dishes were scratch-wounded using a pipette tip and washed twice with PBS. Later, cells were exposed to the indicated treatments for 36 h. Finally, the cells were fixed and crystal-violet-stained. Cell migration into the wound was characterized using an inverted microscope coupled to a digital camera, and processed by ImageJ software (NIH, Bethesda, MD, USA; 1.54c version).

### 2.12. Cell Invasion Assays

A modified Boyden chamber method was used with culture inserts of an 8 μm pore size (Corning, Inc., Corning, NY, USA). Matrigel was added into culture inserts and kept at 37 °C to form a semisolid matrix; after 2 h of starvation, cells were pretreated for 2 h with 12 μM mitomycin-C. Then, the cells were seeded into the upper chamber of the Boyden chamber at 1 × 10^5^ cells/well. Treatments in the DMEM medium were added to the lower chamber. After 48 h of incubation, cells on the lower membrane surface were fixed in methanol for 5 min. Membranes were treated with triton 0.01% and stained with hematoxylin. Later, cells were photographed using an inverted microscope.

### 2.13. Zymography and MMP-9 Characterization by Western Blot

Cell cultures were seeded at a density of 100,000 cells/mL. Under 90% confluency, cellular cultures were incubated under several schemes, and the supernatant medium was collected. Volumes of 40 µL supernatant medium were mixed with 5× sample buffer (0.313 M Tris pH 6.8, 10% SDS, 50% glycerol, and 0.05% bromophenol blue) and loaded on 8% polyacrylamide gels copolymerized with gelatin (1% *w*/*v*). Gels were rinsed twice with 2.5% Triton X-100 and incubated in a development buffer (50 mM Tris–HCl pH 7.4, 10 mM CaCl_2_, and 0.02% NaN_3_) for 40 h at 37 °C. Gels were fixed and stained with 0.25% Coomassie Brilliant Blue G-250. Proteolytic activity was detected as clear bands against the background stain of the unprocessed substrate. β-actin was used as a loading control. Likewise, supernatant media samples were processed by SDS-PAGE. Then, they were transferred to PVDF membranes, and MMP-9 Western blot was performed. As a complementary control, PVDF membranes were Coomassie-stained, generating protein load normalization.

### 2.14. Confocal Microscopy

An LEICA TCS-SP8 confocal scanning biological microscope (LEICA Microsystems Heidelberg GmbH, Nussloch, Germany) was employed to characterize the subcellular localization of dil-LDL (0–50 µg/mL). Cell variants were proliferated to 90% confluence with an initial seeded procedure of 100,000 cells/mL, and treated with dil-LDL for 12–36 h. Later, the culture cells were washed with PBS, and then, the Hoescht probe was added and incubated for 0.5 h at 37 °C. Cells were washed with PBS, fixed with 2% paraformaldehyde for 2 min at 37 °C, and mounted for observation. Different zones were macroscopically registered to depict representative images. Images were recorded according to previous work reported by our group [38].

### 2.15. Exosome (Exs) Purification

Variant B cells were seeded at 100,000 cells/mL on 100 mm dishes and proliferated to 90% confluence. LDL treatment was performed for 24 h (25 µg/mL). Then, treatment was withdrawn, and cell cultures were washed twice with the DMEM medium to eliminate LDL interference. Finally, cell cultures were maintained under incubation for 24 h. An extensive optimization was performed in order to guarantee a high grade of purity and an adequate concentration of exosomes. Each round of purification started with three 100 mm dishes of variant B cells. First, the medium samples were centrifuged twice at 200× *g* for 15 min at 4 °C, followed by further centrifugation of the supernatants at 600× *g* for 30 min. Supernatants were recovered and centrifuged at 2000× *g* for 30 min. Then, using a rotor S50-A 2559, the supernatant was centrifuged at 10,000× *g* for 30 min, and finally at 125,000× *g* for 80 min in a Sorvall MTX 150 ultracentrifuge (Thermo Fisher Scientific, Waltham, MA, USA). This ultracentrifugation step generates the exosome pellet denominated Exs, and the supernatant corresponds to the free medium of exosomes (FM-Exs) used as a control in several experiments. For a relationship of three 100 mm dishes, we used 225 µL of PBS. The quality of exosomes was characterized by the constitutive targets Flotillin-2 (Flot-2) and CD63; in this characterization, we included cell lysates (10 μg) and FM-Exs. Likewise, exosome samples were processed by electron microscopy. The characterization of Flot-2 in samples allowed for the adjustment of the amount of exosome protein for several experiments.

### 2.16. Electronic Microscopy Experimentation

Exosome samples were diluted to 1/3 in PBS prepared with ultrapure H20. Subsequently, the samples were filtered through a 0.22 µm filter. Samples were deposited on Formvar-Carbon TEM grids, copper 300 mesh, and after incubation for 10 min at 25 °C, excess liquid was removed with Whatman paper. Samples were dried for 20 min; images were acquired using JEOL-7800 Prime Field Emission Scanning electron microscopy (Tokyo, Japan) with a scanning transmission electron microscopy detector at 29.5 kV, at a 65,000× magnification. This method was based on a previous work [39]. 

### 2.17. Characterization of Exosome Internalization

Exosomes were isolated according to the specifications in Section 2.15 protocol. Later, purified exosomes (Exs) were stained with a CellMask probe (Thermo Fisher Scientific, Waltham, MA, USA) (2X) (112 µL) for 30 min at 37 °C based on recommendations of the provider. After labeling, Exs-CellMask was diluted in PBS 1X (10 mL) to eliminate the probe by centrifugation at 125,000× *g* for 80 min. The Exs-CellMask pellet was reconstituted in DMEM (200 µL), filtered through a 0.22 µm filter, and used for treatment in cell culture dishes of 20 mm (seeding 100,000 cells). After treatment for 12 h, cells were analyzed in a Cytoflex flow cytometer (Beckman-Coulter), and 20,000 events were registered using 525/40 BP channel and CytExpert software (Supplementary Figure S2).

### 2.18. Lactate Determination

Lactate quantification in supernatant media was based on the protocol of Grist et al., 2018 [40]. A standard curve of 0.093–6 mM was used. This experimentation was carried out in 20 mm dishes under the seeding of 200,000 cells. Upon reaching 90% confluency, cells were treated under several conditions, and lactate concentrations were quantified in supernatant media.

### 2.19. Lipid Droplet Isolation and Cholesterol Quantification

This experimental procedure was carried out in 60 mm dishes with the seeding of 500,000 cells. Variant B cells were processed as reported elsewhere [41]. After LDL treatment, cells were scraped from the plate, transferred to a 2 mL tube, and centrifuged at 2000× *g* for 2 min. The pellet was dissolved in 200 µL of a buffer of 60% sucrose, 10 mM HEPES, and 1 mM EDTA, with a pH of 7.4. After mixing, samples were ice-incubated for 10 min. Next, 800 µL of ice-cold lysis buffer was added and incubated on ice for 10 min. Cells were lysed by 5 passes through a 27-G needle and centrifuged at 100× *g* for 2 min. A mixture of 2 µL of methylene-blue per 1 mL of lysis buffer was prepared; then, 600 µL of this mixture was carefully layered on top of the cell homogenate and centrifuged at 20,000× *g* at 4 °C for 120 min. Tubes were frozen at −70 °C and the dye layer containing lipids was recovered (lipid droplets). The cholesterol content of these fractions was characterized (Spin React, S.A.U., Girone, Spain).

### 2.20. Spheroid Growth Assay

Cell cultures were seeded in 24-well plates coated with 1.5% agarose (400 μL/well). Parental and variant B cells were seeded (8000 cells/well) on the agarose-coated wells. The spheroids were treated for 12 days with LDL (25 µg/mL) and Dox (500 nM), and the treatments were performed at 1, 3, 6, 9, and 12 days. During the experiment, photographs were taken on days 3, 6, and 9 with a camera-coupled microscope. The spheroidal area was analyzed with the ImageJ software. For each time point, the areas of four spheroids were processed.

### 2.21. Statistical Analysis

Data are expressed as the mean ± SD. Statistical analyses were conducted with a one-way ANOVA, and differences among the means were compared with the Bonferroni assay using a significance level of *p* < 0.05, unless otherwise specified. The software used was GraphPad Prism version 6 (San Diego, CA, USA). For the analysis of the expression of proteins by Western blot, a semiquantitative analysis was performed using loading controls with the ImageJ software (Bethesda, MD, USA).

## 3. Results

### 3.1. Parental Cells and Variant B Internalize LDL and Induce Cell Migration

**Figure 1:**
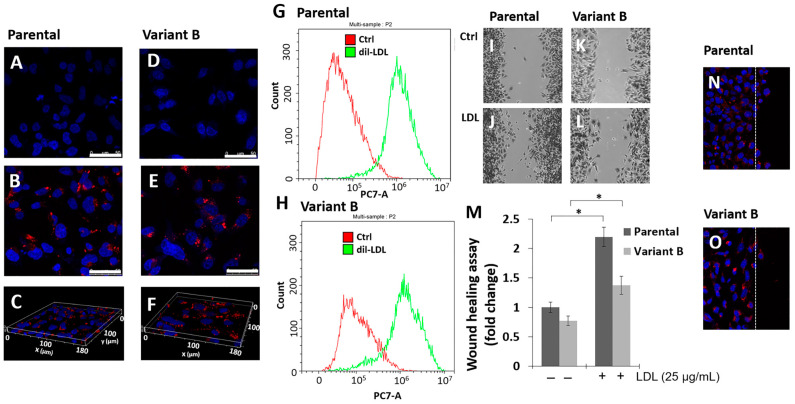
Parental and variant B cells internalize LDL, and cell migration is induced. LDL endocytosis was evaluated by confocal microscopy in parental cells (**A**) and variant B (**D**) controls. Images corresponding to parental (**B**) and variant B (**E**) cells treated with dil-LDL (25 µg/mL); under these conditions, three-dimensional images of parental (**C**) and variant B (**F**) cells. Hoechst was employed for staining cell nuclei; bars correspond to 50 µm. Cytometer analysis in parental (**G**) and variant B (**H**) cells treated with dil-LDL (25 µg/mL). Representative images of the wound healing assay in parental cells corresponding to the control (**I**) and LDL treatment (25 µg/mL) (**J**). For variant B, images corresponded to the control (**K**) and LDL treatment (25 µg/mL) (**L**). (**M**) Wound healing results are expressed as a fold change concerning the control of parental cells. The mean values are presented (*n* = 3, mean ± SD); * *p* < 0.05. Migration front in the wound healing assay in parental (**N**) and variant B (**O**) treated with dil-LDL (25 µg/mL); nuclei were stained with Hoechst.

We previously established several protocols to generate Dox-induced chemoresistant MDA-MB-231 variants (Material and Methods, Section 2.3). Cell cultures were treated with a lethal Dox dose (500 nM) for 24 h, followed by a gradual decreasing dose for 60 days until a dose of 12.5 nM was reached and a recovery period of 30 days. This led to the generation of a variant that will from now on be referred to as variant B [17]. Chemoresistance was coupled with higher concentrations of Dox in the extracellular medium in variant B cells, a phenomenon analyzed later. Considering the LDL effect on chemoresistance, parental (control cells) and variant B cells were treated with dil-LDL (25 µg/mL) for 36 h. The results suggest the induction of dil-LDL internalization in parental and variant B cells, evaluated by confocal microscopy (Figure 1A–F). A slight diminution was registered in variant B (Figure 1E) compared to parental cells (Figure 1B); this characterization was strengthened by three-dimensional analysis corroborating the intracellular LDL localization, respectively (Figure 1C,F). In addition, the dil-LDL endocytic capability was corroborated by flow cytometry (Figure 1G,H). The results showed slightly lower LDL internalization in variant B compared to parental cells.

To gain insights into LDL’s role as a cancer hallmark, we documented the LDL effect on cellular migration in both variants, an increased migration capability under LDL treatment (25 µg/mL) was registered in parental (Figure 1I,J,M) and variant B cells (Figure 1K–M) compared to respective basal conditions. This phenomenon agrees with LDL internalization in both variants, and these responses would be associated with cell lipid accumulation and the activation of specific signal transduction pathways, as we will describe later. To establish the cell features at the migration front [42] and considering the LDL effect, we performed assays to monitor dil-LDL internalization. We did not find significant differences across the analyzed areas in parental (Figure 1N) and variant B (Figure 1O) cells.

### 3.2. LDL Promoted Cell Proliferation, Invasion, and 3D Spheroid Growth

**Figure 2:**
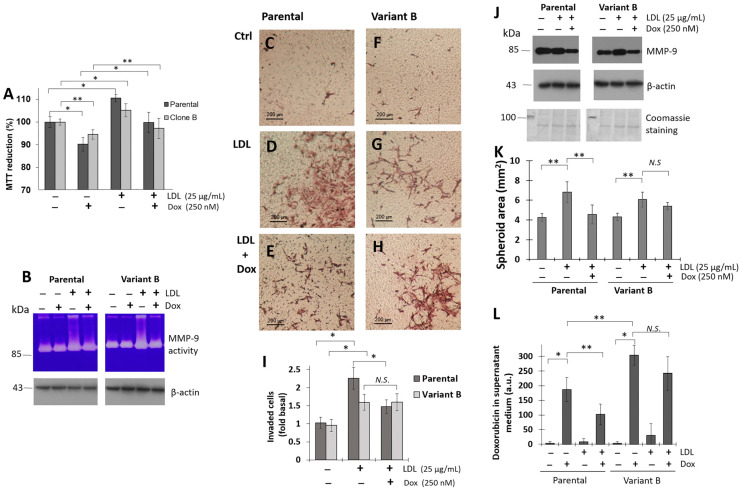
LDL stimulus promotes proliferation, invasion, and 3D spheroid growth. (**A**) Cell viability in parental and variant B cells under LDL (25 µg/mL) and Dox (250 nM) treatments for 48 h. Mean values are presented (*n* = 6, mean ± SD), * *p* < 0.01, ** *p* < 0.05. (**B**) Under the same conditions, MMP-9 activity was analyzed on the supernatant medium. β-actin was used as a loading control. Representative images of invasion experiments; in parental cells, the image corresponds to the control (**C**), LDL treatment (25 µg/mL) (**D**), and LDL and Dox (500 nM) (**E**). For variant B, the images correspond to the control (**F**), LDL treatment (**G**), and LDL-Dox (**H**). Bars correspond to 200 µm. (**I**) Analysis of invasion experiments: graphs are expressed as the fold of control. Mean values are presented (*n* = 3, mean ± SD), * *p* < 0.01. (**J**) Under the same conditions, MMP-9 was characterized by Western blot. Coomassie staining and β-actin were used as a loading control. (**K**) Characterization of changes in the area of spheroids (mm^2^) in parental and variant B cells under the treatment of LDL (25 µg/mL) and Dox (500 nM). Mean values are presented (*n* = 3, mean ± SD), ** *p* < 0.05. N.S.: non-statistical significance. (**L**) Doxorubicin quantification in supernatant media, under Dox (500 nM) and LDL treatment (25 µg/mL). Mean values are presented (*n* = 3, mean ± SD), * *p* < 0.01, ** *p* < 0.05.

The effect of LDL on cancer development pathways was evaluated. In this regard, we registered that LDL treatment (25 µg/mL) induced an increment of cell viability in parental and variant B compared to controls, respectively (Figure 2A); this phenomenon was coupled with the induction of metalloproteinase-9 (MMP-9) activity on supernatant media, wherein increased levels were found in both cell variants (Figure 2B). Reports suggest that the chemoresistant phenotype could be associated with the induction of cellular invasion [43]; therefore, we evaluated this phenomenon by the Boyden chamber method. LDL treatment promoted an increased invasion capacity on parental cells (Figure 2C,D,I) compared with variant B cells (Figure 2F,G,I). Notwithstanding, Dox treatment (500 nM) reduced the invasion capability in parental cells (Figure 2D,E,I), and the inhibition of cellular invasion under Dox treatment was not registered in variant B cells (Figure 2G–I). This cell phenomenon corresponded with the extracellular localization of MMP-9 by Western blot (Figure 2J). 

Considering the influence of LDL on both variants, we focused on the role of LDL in spheroid formation (3D cultures), using a tumor model used to study drug efficacy and resistance to chemotherapy [44]. Based on methodology (Section 2.20), LDL treatment (25 µg/mL) was carried out 24 h after seeding and thereafter every 72 h. Our data suggest an induction of LDL on the growth of the spheroids in both variants with respect to their controls (Figure 2K), although the effect was greater in variant B. Moreover, under Dox treatment, we evidenced an arrest on spheroid growth induced by LDL mainly in parental cells, even with an impact on the spheroid stability generating the disaggregation (Figure 2K). A phenomenon that could be associated with the cellular handling of Dox.

Therefore, Dox quantification was performed in an extracellular medium, and Dox concentrations under basal conditions were lower in parental cells than variant B probably associated with the chemoresistant phenotype (Figure 2L). Nonetheless, LDL treatment reduced Dox levels in the supernatant media of both variants (Figure 2L), although the levels were consistently higher in variant B associated with chemoresistance. The results suggest that LDL treatment can increase cellular invasion, proliferation, and 3D spheroid growth in both parental and resistant cells. Even more, LDL treatment could modify mechanisms such as UPR, as we have reported [7], and protein translation initiation factors such as eIF4A and its regulation could maintain a critical role during chemoresistance.

### 3.3. Implication of the eIF4F Complex for Chemoresistance

**Figure 3:**
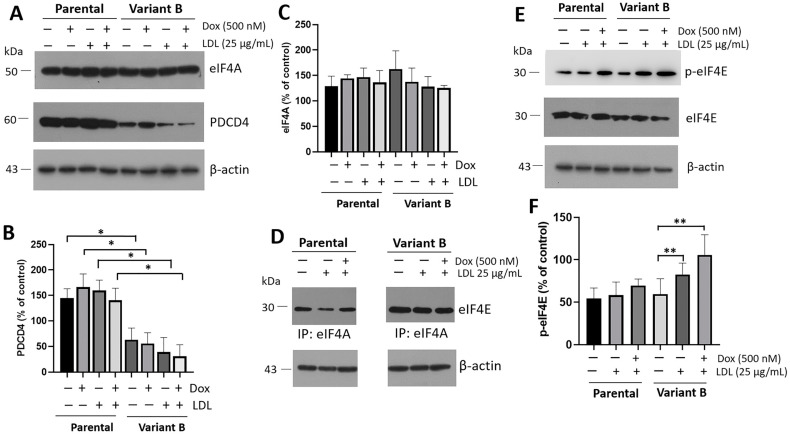
Chemoresistance is connected with the regulation of eIF4F and PDCD4 targets. (**A**) Protein expression of the component of the eIF4F complex, the initiation factor eIF4A, and the negative regulator PDCD4 under the concomitant treatment of LDL (25 µg/mL) and Dox (500 nM). β-actin was used as a loading control. Densitometry analysis of PDCD4 (**B**) and eIF4A (**C**); results are reported as the mean ± SD (*n* = 3) and expressed as % of control; * *p* < 0.005 with respect to the control. (**D**) eIF4A immunoprecipitation under LDL treatment and Dox, detection of eIF4E. Control of β-actin was included. (**E**) eIF4E characterization through the detection of eIF4E-p (Ser-209) and eIF4E (total) in parental and variant B cells under LDL (25 µg/mL) and Dox (500 nM) treatments. (**F**) Densitometry analysis of p-eIF4E; results are reported as the mean ± SD and expressed as % of control; ** *p* < 0.05 with respect to the control.

Cell cultures of parental and variant B were treated under LDL (25 µg/mL) and Dox (500 nM). We registered slightly higher levels of eIF4A in chemoresistant variant B, although the differences were not significant (Figure 3A,C). However, we evidenced that the down-regulation of tumor suppressor gene PDCD4 was associated with Dox chemoresistance. Significantly, our experiments demonstrated the down-regulation of eIF4A’s negative regulator PDCD4 in variant B compared to the parental cells (Figure 3A,B). One of the main mechanisms for PDCD4 down-regulation is mediated by proteasome degradation through the mTOR/p70S6Kα pathway [39], with a potential implication on the eIF4A (Figure 3C) and, therefore, in the eIF4F complex formation. In that regard, the characterization of the eIF4F complex suggests a higher signal in eIF4A–eIF4E interaction in variant B cells (Figure 3D). Indeed, we documented the activation of eIF4E mediated by phosphorylation of Ser-209, which is associated with the translation of the mRNAs involved in tumorigenesis [45], and increased expression of lipid metabolism targets, such as those involved in triglyceride synthesis and lipid droplet formation [46]. This activation is mainly under LDL and combined treatment of LDL (25 µg/mL) plus Dox (500 nM) in variant B cells (Figure 3E,F). 

The eIF4F complex and the activation of p-eIF4E (Ser-209) is associated with a differential effect on the UPR in variant B cells. For instance, tumorigenesis could be associated with the selective translation mediated by the PERK and IRE1 arms of UPR [47]. We analyzed this association, whereby the LDL effect was evaluated by characterizing XBP1s and Nrf2, the targets related to the activation of the IRE1 and PERK pathways, respectively (Supplementary Figure S1). 

Furthermore, the secretion of UPR targets such as chaperones may drive cell communication pathways [17]. Hsp90 and Hsp70 chaperones must modulate cellular signaling mechanisms and contribute in a non-cell-autonomous maintenance of proteostasis [28]; therefore, their secretion may be contained in exosomes under eIF4F activation [48,49]. Even more, LDL stimuli could induce changes in the composition of cargo molecules, based on previous reports indicating that cholesterol and ceramide levels in endosomal membranes influence the content and size of exosomes [27]. 

### 3.4. LDL Treatment Modifies the Chaperone Content in Exosomes Released from Variant B Cells

**Figure 4:**
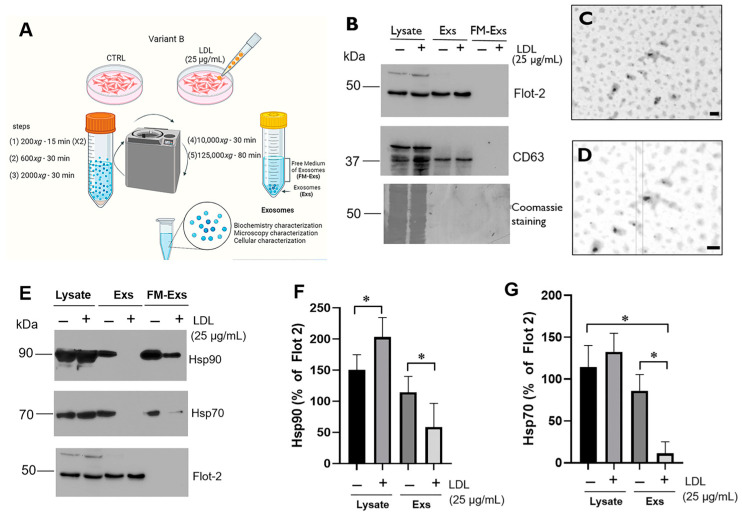
Characterization of exosomes (Exs) isolated from the supernatant media of variant B cells. (**A**) Scheme corresponding to exosome isolation in control and LDL treatment (25 µg/mL). (**B**) Evaluation of flotillin-2 (Flot-2) and CD63 proteins as exosome markers under treatment with LDL (25 µg/mL) for 24 h. Lysate: cellular lysate of variant B; Exs: fraction corresponding to exosomes; FM-Exs: free medium of exosomes. (**C**,**D**) Electron microscopy images corresponding to the Exs samples; the bar indicates 100 nm. (**E**) Identification of the Hsp90 and Hsp70 content in Exs, lysate and FM-Exs samples. Previously, variant B cells were treated with LDL (25 µg/mL) (24 h), and cell cultures were washed and later incubated in the LDL-free medium for 24 h. Flot-2 was used as a loading control. Densitometry analysis of Hsp90 (**F**) and Hsp70 (**G**); results are reported as the mean ± SD (*n* = 3) and expressed as % of Flot-2; * *p* < 0.01, with respect to the specific controls.

LDL’s effect on the exosome content and the induction of the autocrine effect on chemoresistance were analyzed; therefore, we focused on variant B cells. Exosome purification from the supernatant media was carried out through the ultracentrifugation process. Purification of the exosomes released from variant B cells was performed after the LDL treatment (25 µg/mL) for 24 h (Section 2.15). The supernatant media were collected, and exosomes were purified (Figure 4A). In all cases, a vehicle control (PBS) for cellular treatment was evaluated. To analyze the isolation of exosomes, the identification of representative targets such as Flot-2 and CD63 was performed. In this characterization, cellular lysate samples, exosomes (Exs), and free medium of exosomes (FM-Exs) were included (Figure 4B). The identification of the targets Flot-2 and CD63 in exosomes and not in the FM-Exs suggests an adequate isolation from the supernatant medium, in both samples, control and LDL treatment (Figure 4B).

Additionally, exosome samples were visualized using transmission electron microscopy. The images showed a size distribution of 20–100 nm, corresponding to exosomes (Figure 4C,D). Furthermore, XBP1s, a target associated with the IRE1 pathway activation, was evaluated. XBP1s was not detected. However, we do not rule out the possibility that other biomolecules of the UPR pathway may be transported within the exosomes.

Therefore, the analysis of Hsp90 and Hsp70 was performed on the exosome samples obtained from variant B cells under LDL treatment (25 µg/mL). These chaperones are associated with UPR activation and potentially connected with the autocrine effect of exosomes. At first glance, the presence of Hsp90 and Hsp70 in the exosomes was confirmed (Figure 4E–G), and Flot-2 was used as a loading control. Data suggest that LDL treatment induced a significant reduction in the exosome content of Hsp90 (Figure 4E,F) and Hsp70 (Figure 4E,G), while this phenomenon was not reported at intracellular levels (Figure 4E). Additionally, we confirmed the reduction in chaperones in the FM-Exs (Figure 4E), possibly associated with the soluble chaperones.

Chaperone content may function as a distant cell communication signal through receptor modulation, considering the potential chaperone localization in membranes and stimulation of receptors by chaperone-associated exosomes [32,33,50]. A CellMask fluorescent probe was used to label exosomes and evaluate their Exs-Cells Mask internalization. Briefly, purified exosomes (Section 2.15) were stained with the CellMask probe for 30 min, generating Exs-Cells Mask; later, the particles were re-purified. Then, variant B cell cultures were incubated with Exs-Cells Mask, and the +data suggest the cellular internalization (Supplementary Figure S2).

### 3.5. The Impact of LDL on the Exosomes’ Autocrine Mechanism in Variant B Cells

**Figure 5:**
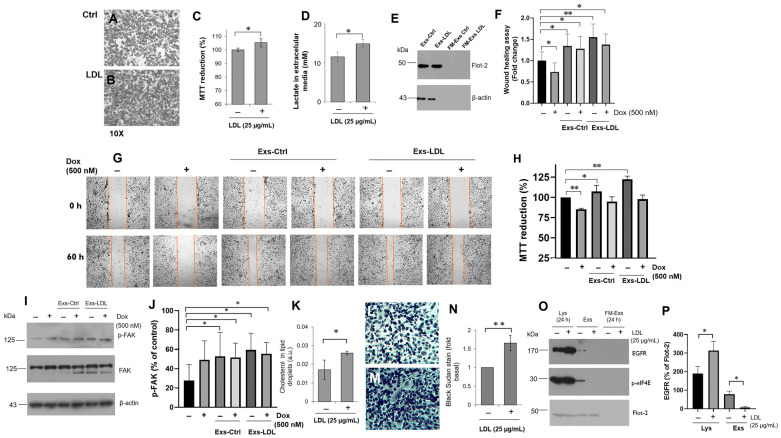
LDL could induce cell proliferation and migration through the autocrine effect mediated by exosomes. Effect of LDL treatment for 24 h (25 µg/mL) on cell density in variant B (**A**,**B**), and the cell proliferation assay with MTT (**C**). (**D**) Extracellular lactate levels under LDL treatment (25 µg/mL) in variant B cells. In panels (**C**,**D**), * *p* < 0.05 with respect to the control. (**E**) Extracellular media were recovered in variant B cells under LDL treatment, and exosomes were isolated and resuspended in PBS (225 µL). Exosome samples were characterized by Flot-2 and β-actin for the normalization of the protein content. (**F**) Cell migration was evaluated through the scratch-wound assay, and exosome treatments were performed 12 h prior to the addition of Dox (500 nM) reaching 60 h of incubation. Mean values are presented (*n* = 3 independent experiments, mean ± SD), * *p* < 0.05, ** *p* < 0.01; (**G**) representative images of optical microscopy showed a greater migration capacity induced by the Exs-Ctrl and Exs-LDL. (**H**) Cell viability evaluation by the MTT assay, mean values are presented (*n* = 6, mean ± SD), * *p* < 0.05; ** *p* < 0.01. (**I**) Characterization of FAK and p-FAK under treatment with Exs-Ctrl and Exs-LDL and concomitant Dox (500 nM). (**J**) Densitometry analysis of p-FAK; results are reported as the mean ± SD (*n* = 3) and expressed as % of control; * *p* < 0.05 with respect to control. (**K**) Cholesterol quantification on lipid droplets in LDL treatment (25 µg/mL). Images corresponding to Black Sudan stain of the control (**L**) and LDL-treated cells under the same conditions (**M**). (**N**) Semi-quantitative analysis of the Black Sudan stain in variant B; graph is expressed as the fold of control. Mean values are presented (*n* = 3, mean ± SD), ** *p* < 0.01. (**O**) Localization of EGFR and p-eIF4E on cellular lysates, Exs, and FM-Exs samples. Flot-2 was used as a loading control. (**P**) Densitometry analysis of EGFR; results are reported as the mean ± SD (*n* = 3) and expressed as % of control; * *p* < 0.05 with respect to the control.

We focused on the characterization of the chemoresistance autocrine mechanism potentiated by LDL treatment in variant B cells, driven by exosomes. In the first instance, we seeded the same amount of cells, and an increase in cellular density after 24 h of LDL treatment (25 µg/mL) was registered (Figure 5A,B). The LDL treatment also led to an increase in the MTT reduction (Figure 5C), along with elevated lactate levels in the extracellular medium (Figure 5D). Indeed, chemoresistance is associated with anaerobic metabolism and increased glycolytic activity [51]. Our results suggest that LDL induces higher metabolic activity in variant B cells.

To evaluate the autocrine effect of exosomes derived from chemoresistant variant B cells, we examined their impact on migratory capability using the scratch-wound assay. Exosomes were purified from variant B cells without treatment, referred to as Exs-Ctrl, and from cells treated with LDL (25 µg/mL), referred to as Exs-LDL, following the previously described methodology (Section 2.15). In this case, based on the abundance of Flot-2 and β-actin in exosomes, we adjusted the volumes to ensure a consistent protein loading (Figure 5E), in this characterization we used FM-Exs as the control. Variant B cells were exposed to exosomes (Exs-Ctrl and Exs-LDL) for 12 h, followed by continued incubation with Dox (500 nM) for 60 h to evaluate its effect on chemoresistance. The results identify an increased migration capability of cells treated with exosomes; particularly, the highest response was found under Exs-LDL incubation. There was a slight reduction in the effect of Dox on cell migration, but it did not reach the observed levels with Dox treatment (Figure 5F,G).

These findings were completed by the MTT proliferation assay, and data showed an induction of cell viability under Exs-Ctrl and Exs-LDL treatments, with the highest proliferation registered under the Exs-LDL (Figure 5H). This finding is consistent with the cellular migration data. Furthermore, exosome treatment prevented the cytotoxicity induced by Dox incubation (500 nM). The results suggest that exosome treatment induced migration and cell viability through an autocrine pathway while protecting against doxorubicin-induced cytotoxicity. This phenomenon was more noticeable under Exs-LDL, which was dependent on metabolic alterations induced by LDL. Likewise, our results suggest the activation of the p-FAK/FAK mechanism (Figure 5I,J) induced by Exs-Ctrl and Exs-LDL. FAK participates as a signaling-protein scaffold for the assembly and subsequent maturation of focal contacts [52]; even maintaining its activation despite treatment with doxorubicin. 

Disorders in cholesterol storage have been linked to alterations in exosome formation and its protein content [53], which could modify exosome-mediated cellular effects. Indeed, in cells treated with LDL, we observed a high cholesterol content in lipid droplets induced by LDL (Figure 5J), as evidenced by the Black Sudan stain assay for neutral lipids (Figure 5K–M), a phenomenon that may be consistent with the activation of eIF4E (Figure 3). The accumulation of intracellular cholesterol may activate mechanisms that impact the exosome content, including chaperones, thereby intensifying the autocrine mechanism of cellular migration and proliferation. Although, the details of this mechanism still need to be elucidated.

To understand the intracellular mechanisms regulated by LDL and their impact on the exosomal protein content, we focused on characterizing p-eIF4E and EGFR. p-eIF4E is associated with the modulation of lipid targets [40] and eIF4F; while EGFR is a constitutively expressed growth factor receptor (Figure 5N). Data suggest that these targets are transported in exosomes with a modulation induced by LDL, leading to a down-regulated localization (Figure 5N,O). These results oppose the expectations, as LDL treatment induced a reduction in the exosome content of EGFR and p-eIF4E, along with the chaperones.

Exosomes represent a distant signal with a specific protein content, which is crucial for the autocrine mechanisms. Importantly, p53 expression is closely connected to modifications in intracellular cholesterol metabolism [6], modulating the expression of critical cholesterol targets such as the suppression of Squalene epoxidase [54], and potentially regulated by chaperone function. Therefore, exosomes could exert a regulatory mechanism.

### 3.6. Autocrine Mechanism Promoted by Exosomes Involved in the p53/Mdm2 Axis and p70S6Ka

**Figure 6:**
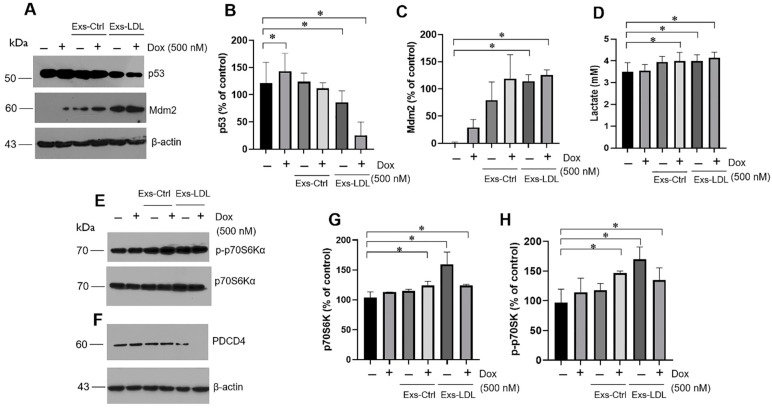
The autocrine mechanism induced by exosomes is mediated through the p53/Mdm2 and p70S6Kα pathways. Changes in p53 are promoted by exosome stimuli (Exs-Ctrl and Exs-LDL) and doxorubicin treatment (500 nM), (**A**) Western-blot of p53 and Mdm2, under treatment with Exs-Ctrl and Exs-LDL (2.5% *v*/*v*) for 36 h. Densitometry analysis of p53 (**B**) and Mdm2 (**C**); results are reported as the mean ± SD (*n* = 3) and expressed as % of control; * *p* < 0.05 with respect to the control. β-actin was used as a loading control. (**D**) Levels of lactate in supernatant media under the same conditions. Effect of exosome treatment (Exs-Ctrl and Exs-LDL) (2.5% *v*/*v*) and doxorubicin (500 nM) on the regulation of p70S6Kα (**E**) and PDCD4 (**F**). Densitometry analysis of p70S6Kα (**G**) and p-p70S6Kα (**H**), results are reported as the mean ± SD (*n* = 3) and expressed as % of control; * *p* < 0.05 with respect to the control.

Our results present evidence of the LDL effect on the aggressiveness of cancer cells, particularly in chemoresistant cells, mediated by exosome signaling. This signaling pathway may have an impact on the critical pathway p53, which is a canonical target during cancer pathogenesis, even more, in human breast cancer MDA-MB-231 cells with high levels of mutant p53 [55]. The results revealed a reduction in p53 expression under exosome treatment, with a significant change observed in the Exs-LDL treatment, and even in concurrent Dox incubation (500 nM) (Figure 6A,B). This response was associated with an increase in Mdm2 expression, one of the main negative regulators of p53, primarily induced by Exs-LDL treatment (Figure 6A,C).

The results suggest that the expression of p53 and Mdm2 is modified under an autocrine exosome treatment in variant B cells, leading to increased cell proliferation and migration (Figure 5). This phenomenon may be correlated with alterations in energetic metabolism. Under exosome treatment (Exs-Ctrl and Exs-LDL), the results showed an increase in lactate release (Figure 6D). Furthermore, the cellular effects induced by exosomes were regulated through the activation of the canonical p70 ribosomal protein kinase S6 (p70S6Kα) signaling pathway (Figure 6E), as well as the down-regulation of PDCD4 (Figure 6F). Notably, stimulation with exosomes led to the activation of the mTOR/p70S6Kα pathway, with higher activation registered in the Exs-LDL treatment (Figure 6E,G,H). 

## 4. Discussion

Chemoresistance is often reported in cancer patients with underlying obesity and dyslipidemias [8,9,10]. One of the triggering factors implicated in this phenomenon is an increased plasmatic LDL concentration. Based on our results, LDL could represent a condition for the induction of proliferation, migration, and invasion in a model of TNBC cells. In this work, we performed the LDL isolation and fluorescent labeling (dil-LDL), and the results proved LDL internalization in both parental and variant B cells. Our results indicated that LDL protects parental and variant B cells from the cytotoxic effects of doxorubicin, promoting cell migration in both variants (Figure 1). An increment in cellular invasion coupled to MMP-9 activity in the extracellular medium was registered (Figure 2), although Dox treatment induced a reduction in this process only in parental cells. Therefore, our evidence suggests a potential role of LDL cholesterol overload in oncogenic processes [17]. This proposal was supported by the influence of LDL stimuli in the spheroid growth assay mainly in variant B cells, and the extended stability registered in variant B regarding the parental spheroids under Dox treatment (Figure 2).

Moreover, evidence shows that the mechanisms associated with obesity and dislipidemias could decrease the efficacy of doxorubicin in TNBC tumors [56,57]. For instance, pathways such as de novo fatty acid synthesis and lipolysis were increased in breast tumor tissue in a diet-induced study, possibly conferring a more saturated lipid cell membrane associated with protecting cancer cells from the cytotoxic effects of chemotherapeutic agents [57]. Other authors have reported an implication for migration process associated with elevated LDL concentration treatment, activating the Ras/Raf-1/MEK/ERK pathway [58]. Therefore, our results suggest the influence of LDL on the effectiveness of this pharmacological treatment.

Moreover, we characterized the eIF4F complex; data indicate a higher signal in the interaction eIF4A-eIF4E in variant B, which is reflected in a higher response in the activation of p-eIF4E under treatment with LDL and Dox (Figure 3). Indeed, we have extensively analyzed the p-eIF4E regulation in a previous review [59]. This response agrees with the chemoresistance behavior in variant B. On the other hand, oxidized low-density lipoprotein (Ox-LDL) treatment induced the down-regulation of ribosomal and translation initiation proteins in retinal pigment epithelial cells [60]. 

The impact of LDL hypercholesterolemia should be widespread and other mechanisms triggered by LDL must be involved, probably modifying cellular communication processes mediated by exosomes. Indeed, reports indicated that exosomes and their content can function as cell-to-cell communicators, playing a role in cancer progression [61]. Therefore, the exosome isolation derived from variant B cells was carried out, and their characterization corroborated features regarding size and protein markers, and through exosome fluorescent labels, cell internalization was confirmed (Figure 4, and Supplementary Figure S2). Critically, exosome treatment was associated with autocrine cellular responses, including proliferation, migration, and metabolic changes. A metabolic effect on lactate release and cell migration was particularly evident in the Exs-LDL treatment group, which agrees with the report of Zhao et al. [62]. This cellular behavior, known as the Warburg effect, promotes cellular proliferation and subsequently stimulates lactate production [63].

LDL treatment resulted in a specific profile of exosome secretion in chemoresistant variant B cells, triggering an autocrine phenomenon that induced cellular proliferation, migration, and a slight increase in chemoresistance. These results suggest a new feature in the mechanisms promoted by LDL. In this regard, evidence suggests that exosomes maintain signaling pathways in the tumor cell microenvironment [23]. Our results showed an autocrine effect induced by exosomes on chemoresistant variant B cells, a phenomenon enhanced by LDL treatment. Indeed, cholesterol and ceramide levels in cellular membranes influence the content and size of exosomes [27]. Based on our results, cholesterol LDL impacts intracellular cholesterol storage (Figure 5L–N). 

Exosomes could affect other cellular processes. An association between FAK and chemoresistance has also been proposed [64]. In this regard, we reported the activation of FAK under the stimulation of Exs-Ctrl and Exs-LDL, even with Dox treatment (Figure 5I,J). For instance, in ovarian cancer cells, increased FAK activity and protein expression were associated with greater tumor aggressiveness and chemotherapy resistance, leading to the expression of DNA repair genes and Myc [64]. These conditions play an indispensable role in tumor biology and are involved in tumorigenesis, progression, and response to treatment, mediated by exosomes. 

Likewise, the protein targets of p70S6Kα signaling can be part of the mechanisms involucrated during cancer development (Figure 6). For instance, in renal cell carcinoma, fluvastatin inhibits the kinase p70S6Kα, accompanied by an increase in the expression of PDCD4 [65] and the formation of the eIF4E-binding protein-eIF4E complex, inducing apoptosis. In our case, after treatment with Exs-LDL, we observed a noticeable increase in p70S6K and a decreased expression of PDCD4, even in the combination with Dox treatment, which is associated with chemoresistance promoted by exosomes (Figure 6E–H), suggesting an increase in protein synthesis and cell proliferation induced by Exs-LDL treatment. This was in accordance with the reduced expression of PDCD4 induced by LDLs.

Likewise, the response by exosomes released from variant B cells under LDL treatment was associated with the down-regulation of p53. This mechanism was coupled with changes in the regulator Mdm2 (Figure 6). In this case, the ubiquitin (Ub)–proteasome system plays a pivotal role in regulating p53 protein stability and activity; p53 is ubiquitinated and destabilized by Mdm2 and other Ub E3s. Since p53 aggregation can be induced by Hsp90 [33], this phenomenon could impact p53 levels regulated by the exosome treatment. This condition is relevant, considering that mutant p53 gain-of-function could interact with other targets and regulate cancer cell transcriptional programs [66]. Moreover, Hsp70 and Hsp90 chaperones could contribute to regulation of the conformation of p53 DNA-binding domain and several p53 cancer variants [67]. 

The protein content of exosomes could be dynamic and dependent on the metabolic status. In this regard, the transport of chaperones in exosomes reflects cellular homeostasis. Our results demonstrated the presence of Hsp90 and Hsp70 in exosomes, and we found their down-regulation induced by LDL treatment. In this regard, evidence suggests the localization of Hsp90α on the external surface of tumor-secreted exosomes [15]. Moreover, CD91+ scavenger receptor signaling stimulated by extracellular HSPs could be involved in cancer progression [32]. However, this immunostimulatory function should be considered only the tip of the iceberg regarding the different functions of Hsp90. Hsp90 activity is connected to VEGF, a process related to the induction of angiogenesis [49], and importantly, a mechanism mediated by Exs-LDL (Supplementary Figure S3). Hsp90 overexpression is a factor of tumorigenesis [30] and it is beginning to be associated with chemoresistance [68]. Co-chaperones such as Hsp70 are necessary for Hsp90 activity [30].

Considering the significant decrease in Hsp90 and Hsp70 induced by LDL treatment in both Exs and FM-Exs, HSPs have also been connected to the homeostasis of cellular cholesterol metabolism [69]. For instance, H90 inhibitors reduced cholesterol storage in Niemann–Pick-type C1 mutant fibroblasts [70]. In addition, the autocrine loop hypoxia HSP90–LDL receptor-related protein 1 (LRP1) is associated with migration [71]. Moreover, 3-hydroxy-3-methyl-glutaryl-coenzyme A reductase inhibition, a critical enzyme for cholesterol biosynthesis, led to an increase in glycolysis via regulated HSP90 expression levels, inducing tumor growth acceleration [72]. Although we have not elucidated the mechanism, we propose that the down-regulation of Hsp must be a mechanism to maintain cellular homeostasis and cholesterol balance.

This condition allows us to reconsider the complexity and arrangement of these biomarkers in exosomes. Furthermore, chaperone signaling via exosomes could contribute to maintaining protein homeostasis at the organismal level [28]. However, under oncogenic conditions, increased cellular LDL cholesterol must have a predominant factor. The specific content of Hsp90-Hsp70 may function as a biomarker for autocrine and paracrine signaling, given their association with a high cellular expression of Hsp90. Considering their increased expression levels in several tumors and their role in multiple signaling pathways [13], the function of chaperones in maintaining cellular homeostasis and autocrine pathways may be a critical factor in the development of cancer cells. Therefore, exosomes may serve as communication factors that promote chemoresistance and oncogenic mechanisms.

## 5. Conclusions

Our findings suggest that LDL treatment promoted increased cell proliferation, migration, invasion, and spheroid growth in parental and variant B cells (doxorubicin-resistant), which are models of TNBC cells. Moreover, autocrine stimulation with exosomes released under LDL treatment in variant B cells may be associated with increased proliferation, migration, and metabolic alterations; the specific Hsp90/Hsp70 content is possibly an inducer factor. These findings represent innovative approaches to understand the factors contributing to chemoresistance and the promotion of aggressive oncogenic phenotypes.

## Data Availability

Data are contained within the article.

## Supplementary Materials

The following supporting information can be downloaded at: https://www.mdpi.com/article/10.3390/biomedicines12040742/s1, Figure S1: Protein expression of the targets XBP1s (A) and Nrf2 (B) on cellular lysates of parental and Variant B cells, under the concomitant treatment of LDL (25 μg/mL) and Dox (500 nM); Figure S2: Characterization of exosome internalization in variant B cells; Figure S3: Regulation of VEGF and p-eIF4E under the treatment with Exs-Ctrl and Exs-LDL (2.5% *v*/*v*) and concomitant incubation with Doxorubicin (Dox) (500 nM) for 36 h.
