# Supplementary material for: Low-Density Lipoproteins Increase Proliferation, Invasion, and Chemoresistance via an Exosome Autocrine Mechanism in MDA-MB-231 Chemoresistant Cells
Source: Biomedicines. 2024 Mar 27;12(4):742. doi: 10.3390/biomedicines12040742 (PMC11048396; doi:10.3390/biomedicines12040742)

Low-Density Lipoproteins Increase Proliferation, Invasion, and Chemoresistance via an Exosome Autocrine Mechanism in MDA-MB-231 Chemoresistant Cells

César Y. Castañeda-Sánchez [1,2], Brenda Chimal-Vega [1,2], Roberto León-Gutiérrez [1,2], Adrián Ernesto Araiza-Robles [1,2], Nicolás Serafín-Higuera [3], Angel Pulido-Capiz [1,2], Ignacio A. Rivero [4], Raúl Díaz-Molina [1,2], Manuel Alatorre-Meda [5], Eustolia Rodríguez-Velázquez [6,7] and Victor García-González [1,2,]*

[1] Departamento de Bioquímica, Facultad de Medicina Mexicali, Universidad Autónoma de Baja California, Mexicali 21000, Mexico; yahel.castaneda@uabc.edu.mx (C.Y.C.-S.); brenda.chimal@uabc.edu.mx (B.C.-V.); roberto.leon@uabc.edu.mx (R.L.-G.); adrian.araiza@uabc.edu.mx (A.E.A.-R.); pulido.angel@uabc.edu.mx (A.P.-C.); rauldiaz@uabc.edu.mx (R.D.-M.)
[2] Laboratorio Multidisciplinario de Estudios Metabólicos y Cáncer, Universidad Autónoma de Baja California, Mexicali 21000, Mexico
[3] Facultad de Odontología Mexicali, Universidad Autónoma de Baja California, Mexicali 21000, Mexico; nserafin@uabc.edu.mx
[4] Centro de Graduados e Investigación en Química, Tecnológico Nacional de México, Instituto Tecnológico de Tijuana, Tijuana 22510, Mexico; irivero@tectijuana.mx
[5] Centro de Graduados e Investigación en Química-Grupo de Biomateriales y Nanomedicina, CONAHCYT-Tecnológico Nacional de México, Instituto Tecnológico de Tijuana, Tijuana 22510, Mexico; manuel.alatorre@tectijuana.edu.mx
[6] Facultad de Odontología, Universidad Autónoma de Baja California, Tijuana 22390, Mexico; eustolia.rodriguez@uabc.edu.mx
[7] Centro de Graduados e Investigación en Química-Grupo de Biomateriales y Nanomedicina, Tecnológico Nacional de México, Instituto Tecnológico de Tijuana, Tijuana 22510, Mexico
* Correspondence: vgarcia62@uabc.edu.mx; Tel.: +52-686-557-1622 (ext. 45309)

Sup. Fig. S1 Protein expression of the targets XBP1s (**A**) and Nrf2 (**B**) on cellular lysates of parental and Variant B cells, under the concomitant treatment of LDL (25 µg/mL) and Dox (500 nM). β-actin was used as a loading control.

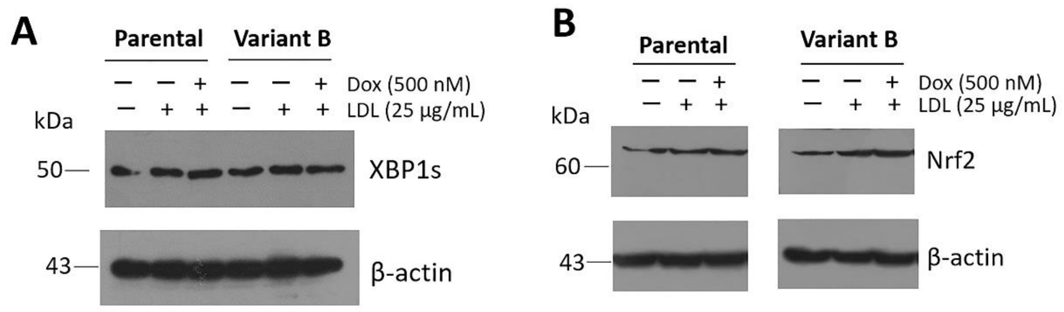

Sup. Fig. S2. Characterization of exosome internalization in variant B cells. **A**) Scheme corresponding to exosome isolation and staining. Purified exosomes (Exs) were stained with a CellMask probe generating Exs-CellMask. **B**) Variant B cells were treated with Exs-CellMask for 12 h and analyzed in a Cytoflex flow cytometer (20,000 events).

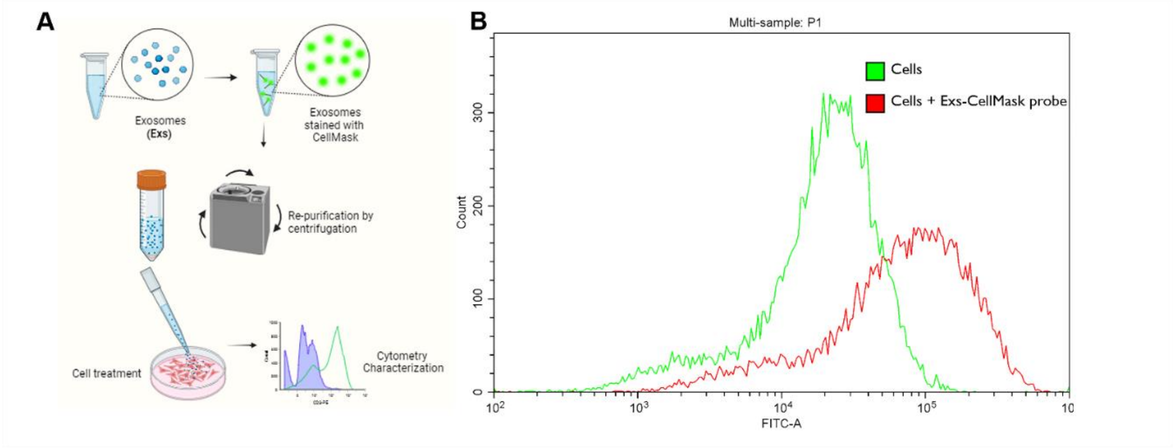

Sup. Fig. S3. Regulation of VEGF and p-eIF4E under the treatment with Exs-Ctrl and Exs-LDL (2.5% v/v) and concomitant incubation with Doxorubicin (Dox) (500 nM) for 36 h.). β-actin was used as a loading control.

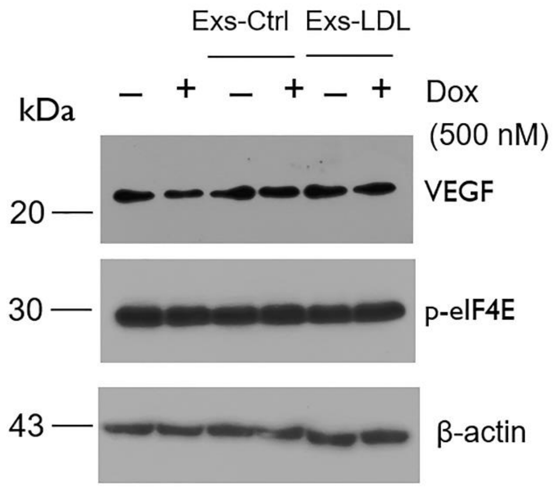

Supplement: Supplementary file 1 [file biomedicines-12-00742-s001.zip › biomedicines-2814960-supplementary.pdf]
